# Influence of Surface Treatments and Thermocycling on Wettability, Roughness, and Bond Strength of Lithium Disilicate CAD/CAM Ceramics

**DOI:** 10.3390/ma18214831

**Published:** 2025-10-22

**Authors:** Serpil Demiroğlu, Salim Ongun

**Affiliations:** Department of Prosthodontics, Faculty of Dentistry, Final International University, Nicosia 99010, Turkey; serpil.ciftcioglu@final.edu.tr

**Keywords:** CAD/CAM ceramics, surface treatment, thermal cycle, surface roughness, bond strength

## Abstract

This study evaluated the effects of surface treatments and thermal aging on the surface properties and bonding performance of three CAD/CAM lithium disilicate ceramics: Amber Mill, Vita Suprinity, and IPS e.max CAD. A total of 150 specimens were divided into five groups: control, hydrofluoric acid etching, airborne-particle abrasion, and Er,Cr:YSGG laser irradiation at 2 W and 3 W. Half of the samples underwent thermocycling (10,000 cycles, 5–55 °C). Surface roughness, water contact angle, and microshear bond strength were measured, and failure modes were analyzed. Ceramic type and surface treatment significantly affected outcomes. Amber Mill generally exhibited higher roughness, while IPS e.max CAD achieved the greatest bond strength. Hydrofluoric acid etching consistently enhanced bonding in Vita Suprinity and IPS e.max CAD, whereas 2 W laser irradiation improved bond strength without notably changing roughness. In contrast, 3 W laser treatment increased contact angle, reducing wettability. Thermocycling raised bond strength in control and sandblasted groups but overall increased contact angle. Adhesive failures predominated, though cohesive and mixed failures became more common after aging. Overall, ceramic type, surface treatment, and thermal aging interactively influenced adhesion. Hydrofluoric acid etching remains the most effective treatment, while low-power laser irradiation offers a promising, less invasive alternative.

## 1. Introduction

The rapid progression of dental materials and CAD/CAM technology in dentistry in recent decades has considerably broadened the array of restorative choices accessible to dentists [1]. All-ceramic restorations have become particularly preferred among these. Lithium disilicate is notable for its exceptional optical properties, great translucency and mechanical properties [2].

Lithium disilicate glass ceramics are prominent in restorative dentistry due to their excellent mechanical properties, esthetic value, and reliable strength when used with adhesive resin cements [3,4]. Blocks such as IPS e.max CAD, Vita Suprinity and Amber Mill are produced using CAD/CAM technologies which offers high flexural strength (~360–400 MPa) and translucency, making them suitable for indirect restorations like inlays, onlays, veneers and crowns [5,6].

Nano lithium disilicate (NLD; Amber Mill, Hass, Gangneung, Republic of Korea), zirconia-reinforced lithium silicate (ZLS; Vita Suprinity, Vita Zahnfabrick, Bad Säckingen, Germany) and lithium disilicate (LDS; IPS e.max CAD, Ivoclar Vivadent, Schaan, Liechtenstein) ceramics represent three advanced CAD/CAM glass-ceramic materials distinguished by their unique microstructural features and clinical advantages [7]. NLD demonstrates customizable translucency via different firing protocols—with minimal impact on flexural strength—offering both esthetic versatility and reliable mechanical performance [8,9]. ZLS ceramics exhibit improved mechanical properties—such as enhanced fracture toughness and flexural strength—compared to conventional lithium disilicate, while maintaining excellent optical characteristics [10]. Lastly, LDS glass ceramics continue to be a benchmark material, supported by narrative reviews affirming its consistently high flexural strength and clinical reliability among chairside CAD/CAM glass-ceramics [6].

Permanently bonding resin luting agents to lithium disilicate ceramics is necessary to provide long-term clinical success for ceramic restorations. Various surface treatments, including hydrofluoric acid etching, alumina air abrasing, and laser irradiation, have been proposed to improve micromechanical retention and surface wettability [11,12]. Hydrofluoric acid etching remains the norm for creating a retentive surface through selective glassy matrix dissolution [13]. Alternatively, aluminium oxide (Al_2_O_3_) airborne particle etching can be employed to create surface roughening by a mechanical process [14]. More recently, Er,Cr:YSGG laser irradiation has been found to be a contactless method for surface modification of ceramics and offers controlled ablation and surface conditioning with minimal heat damage [15].

A few studies have examined laser-irradiated lithium disilicate surfaces. For instance, Alkhudhairy et al. reported that 4.5 W Er,Cr:YSGG laser treatment gave shear bond strengths comparable to those from hydrofluoric acid etching [11]. Noor Azad Mohammed et al. showed Er,Cr:YSGG and CO_2_ lasers significantly increase lithium disilicate surface roughness compared to untreated controls [16]. However, the influence of laser parameters—i.e., energy output—on surface properties and bond strength is variable, with higher setting power at times lowering quality of bond by inducing excessive crystal damage [17].

Thermal aging caused by thermocycling simulates oral environment temperature fluctuations and can subject potential hydrolytic degradation and mechanical wear in the region of the resin–ceramic bond [18].

Although numerous detailed investigations exist on the influence of surface treatments on IPS e.max and other ceramic materials, comparative data involving Amber Mill, Vita Suprinity, and IPS e.max CAD, especially under various laser energy levels and after thermal aging, are limited. Therefore, the aim of this study was to:

1. Analyze and compare the effect of five surface treatments (control, hydrofluoric acid etching, air-borne particle abrasion, Er,Cr:YSGG laser irradiation at 2 Watt, and 3 Watt) on surface roughness, wettability, and microshear bond strength (µSBS) of Amber Mill, Vita Suprinity, and IPS e.max CAD.
2. Evaluate the impact of thermocycling (10,000 cycles between 5 °C and 55 °C) on the durability of resin–ceramic bonds.

The null hypotheses of this study were that (1) different surface treatments will not affect the microshear bond strength, surface roughness and wettability of lithium disilicate CAD-CAM ceramics, (2) the type of lithium disilicate CAD-CAM ceramic material will not affect the microshear bond strength, surface roughness and wettability and that (3) thermal aging will not affect the microshear bond strength, surface roughness and wettability.

The motivation for this study stems from the ongoing challenge of achieving durable adhesion between resin cements and CAD/CAM lithium disilicate ceramics, particularly after thermal aging. The research focused on three lithium disilicate-based ceramics—Amber Mill, Vita Suprinity, and IPS e.max CAD which differ in composition and microstructure. The novelty of this study lies in the inclusion of Amber Mill, a newly developed material with an adjustable crystalline phase, allowing its performance to be compared with established ceramics under standardized conditions.

## 2. Materials and Methods

### 2.1. Materials

Three types of CAD/CAM lithium disilicate-based ceramics were used in this study: Amber Mill (HASS, Gangneung, Republic of Korea), Vita Suprinity (VITA Zahnfabrik, Bad Säckingen, Germany), and IPS e.max CAD (Ivoclar Vivadent, Schaan, Liechtenstein). The chemical compositions and manufacturers’ information are listed in Table 1.

### 2.2. Specimen Preparation

**Figure 1:**
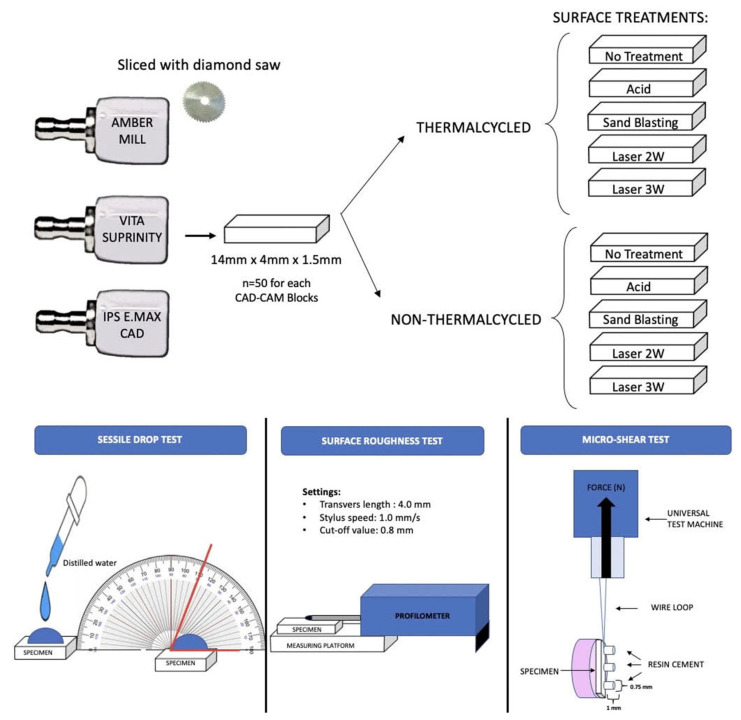
Experimental design.

The experimental design of this study is depicted in Figure 1. From each Lithium Disilicate CAD-CAM block, 50 specimens (in total 150) were wet-sliced (IsoMet 5000, Automatic Precision Sectioning Machine, Buehler Ltd., Seoul, Republic of Korea) into rectangular plates (14 mm × 4 mm × 1.5 mm). Only one surface of each plate was wet polished with 400-grit followed by 600-grit abrasive silicon carbide paper with a grinder and polisher device (MetaServ 250, Buehler Ltd.) for 15 s. A digital calliper was used to arrange the final thickness to 1 ± 0.01 mm of each plate. All specimens were washed with distilled water and air-dried to remove any surface waste and subjected to the crystallization firing process, according to 101 the manufacturer’s recommendations. A total of 50 specimens of AM, 50 specimens of vs. 102 and a total of 50 specimens from IPS was obtained.

### 2.3. Surface Treatments

The specimens were randomly divided into five groups according to the applied surface conditioning method: control (no treatment), hydrofluoric acid etching, airborne particle abrasion, and Er,Cr:YSGG laser irradiation at two different power settings (2 W and 3 W). For the hydrofluoric acid group, 9% Buffered Hydrofluoric acid porcelain etch (UltradentTM) was applied to one surface of each specimen for 20 s, then rinsed and dried. Airborne particle abrasion was performed with 50 μm Al_2_O_3_ particles to one surface of each specimen under 2 bar air pressure at a 10 mm distance for 30 s. For the laser groups, irradiation was performed with an Er,Cr:YSGG laser (Waterlase MD; Biolase Technology Inc., Irvine, CA, USA) to one surface of each specimen using a MG6 Sapphire tip (Biolase Technology Inc.) at a hard tissue mode, with a 2 Watt energy level to one group and 3 Watt energy level to the other group, repetition rate of 10 Hz and a pulse duration of 140 μs with 55% of water and 65% of air, from a distance of 10 mm for 30 s, ensuring that the entire surface was evenly irradiated.

### 2.4. Thermal Aging (Thermocycling)

All specimens of each group were separated into different containers and each group was further divided into two groups; half of the specimens were randomly chosen and 125 thermally aged using a thermocycling machine (Thermocycler 1100/1200, SD 171 Mechatronik, Feldkirchen-Westerham, Germany) and the other half was left in distilled water for 24 hrs. The randomly chosen specimens were thermally aged for 10,000 cycles that corresponds to a year of clinical function in a distilled water bath that ranged from 5 °C to 55 °C with a 30 s dwell time [18]. To compensate for water evaporation overnight, the water tanks were refilled with ~1 L of distilled water each day. The thermocycling machine was monitored on a regular basis to ensure that specimens moved consistently between the tanks, that there was sufficient water available, and that the temperatures in each water bath were stable.

### 2.5. Surface Characterization

#### 2.5.1. Surface Roughness (Ra)

Surface roughness (Ra) was measured using a contact profilometer (Mitutoyo SJ-210, Tokyo, Japan) with a 0.8 mm cut-off length, 1.0 mm/s stylus speed and 4.0 mm transverse length. Three measurements were taken per specimen, and the mean value was recorded.

#### 2.5.2. Water Contact Angle (WCA)

Wettability was determined by measuring the water contact angle using the sessile drop method with a goniometer (KRÜSS DSA25, Hamburg, Germany). A 2 µL droplet of distilled water was placed on each surface, and the angle was recorded after 5 s at room temperature.

#### 2.5.3. Microshear Bond Strength (µSBS)

For the microshear bond strength test, all specimens were first treated with a silane coupling agent (Clearfil Ceramic Primer Plus, Kuraray Noritake Dental Inc., Tokyo, Japan) for 60 s and gently air-dried prior to resin cement application. Then resin samples were prepared on rectangular ceramic specimens. Three Tygon tubes (2 mm in height, 1 mm in inner diameter) were positioned on the surface of each plate. A dual-cure resin luting agent (PANAVIA SA Cement Universal) was dispensed into the tubes using a mixing syringe and excess cement was cleaned around the tubes before light curing to ensure a well-defined bonding area. Each Tygon tube was light-cured for 40 s. The specimens were then stored in distilled water at room temperature for 24 h. After storage, the Tygon tubes were carefully removed and all specimens were examined under magnifying loupes to identify any defects in the resin cement cylinders, including air bubbles, excess resin beyond the bonding limits, or misfit between the cylinders and any incomplete or failed samples were remade. This procedure yielded a total of 150 resin samples for each material. Microshear bond strength testing was performed using a universal testing machine (EZ-test-500 N, Shimadzu, Kyoto, Japan). The 150 rectangular specimens carrying 450 resin cylinders were fixed to the device with a cyanoacrylate adhesive (Zapit, Dental Ventures of America; Corona, CA, USA). A 0.2-mm diameter metal wire was looped around each resin cylinder, and shear force was applied at a crosshead speed of 1 mm/min until failure occurred.

The fracture load was recorded, and the microshear bond strength (µSBS) was calculated according to the equation μSBS = F/A, where F denotes the failure load (N) and A represents the bonded surface area (mm^2^). The value was expressed in megapascals (MPa), following the guidelines of ISO 29022:2013 for adhesion testing in dentistry [19].

### 2.6. Failure Mode Analysis

After testing, debonded surfaces were examined under a stereomicroscope (Leica S8 APO; Leica Microsystems GmbH, Wetzlar, Germany) at a 40× magnification to determine the failure mode. Debonded surfaces were classified as: (1) adhesive, when separation occurred at the bonding interface without resin remnants; (2) cohesive, when failure occurred within the resin; and (3) mixed, when both adhesive and cohesive failures were present.

### 2.7. Statistical Analysis

Power analysis was conducted using G*Power software (Version 3.1.9.3, University of Düsseldorf, Düsseldorf, Germany) to determine the required sample size. With 80% power and a 95% confidence level at α = 0.05, the minimum sample size was calculated as five per group. Accordingly, five specimens were prepared for each test group to achieve the desired statistical power.

Statistical analysis was performed using SPSS software (Version 19; SPSS Inc., Chicago, IL, USA). Because the data did not meet the assumptions of normality and homogeneity, analysis of variance (ANOVA) was not used. Instead, nonparametric analyses were applied. Kruskal–Wallis tests were conducted on three variables—material, surface treatment, and aging—and multiple Mann–Whitney tests were used for post hoc comparisons between groups. The significance threshold was adjusted to α = 0.001 to account for multiple comparisons.

## 3. Results

The statistical analysis was unable to confirm the assumptions required for analysis of variance (ANOVA) due to lack of homogeneity (Levene’s test *p* < 0.05) and normality (Kolmogorov–Smirnov test *p* < 0.05); therefore, ANOVA was not used in this study. Instead, nonparametric analyses were applied. Kruskal–Wallis tests were conducted on three variables: material, surface treatment, and aging. Multiple Mann–Whitney tests were used for post hoc comparisons between groups. To account for multiple comparisons, the significance threshold was adjusted to *α* = 0.001.

In this study, five different surface treatments (control, acid etching, sandblasting, 2 W and 3 W Er,Cr:YS) were applied to three different CAD/CAM lithium disilicate ceramics (AM, VS, and IPS) and were evaluated for their effects on surface roughness, water contact angle, and microshear bond strength following five different surface treatments (control, acid etching, sandblasting, 2 W and 3 W Er,Cr:YSGG laser) and thermal cycling. The findings are presented under separate headings for each parameter.

### 3.1. Surface Roughness (Ra)

The surface roughness values of the three computer-aided design/computer-aided manufacturing (CAD/CAM) lithium disilicate-based ceramics following different surface treatments and thermal aging are summarized in Table 2. Amber Mill generally exhibited higher arithmetic mean surface roughness values than Vita Suprinity and IPS e.max CAD. Hydrofluoric acid etching on Vita Suprinity resulted in the lowest surface roughness. The 2-Watt laser irradiation produced relatively stable surface characteristics, while the 3-Watt laser irradiation caused more pronounced surface alterations in certain groups.

### 3.2. Water Contact Angle (WCA)

As shown in Table 3, the water contact angle values generally increased after thermocycling, indicating a reduction in surface wettability. Hydrofluoric acid etching produced slightly higher or comparable contact angles than the non-aged condition, especially in Amber Mill, suggesting that selective glass-matrix dissolution may modify surface chemistry and energy rather than uniformly enhancing wettability. The 3 W laser groups exhibited the highest contact angle values across all ceramics, corresponding to the lowest surface wettability, whereas the 2 W laser produced intermediate results.

### 3.3. Microshear Bond Strength (µSBS)

Microshear bond strength results are presented in Table 4. The highest bond strength values were observed with hydrofluoric acid etching and laser irradiation—especially 2 W laser application—in the IPS e.max CAD and Amber Mill groups. After thermal cycling, microshear bond strength values increased in many groups. Overall, the findings indicate that ceramic type, surface treatment, and thermal cycling have distinct and interactive effects on all three evaluated parameters.

### 3.4. Failure-Mode Distribution

**Figure 2:**
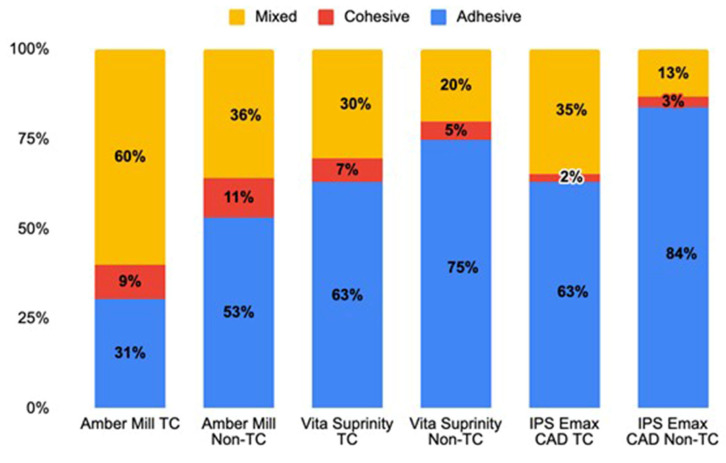
Failure modes of each group after microshear bond strength test.

**Figure 3:**
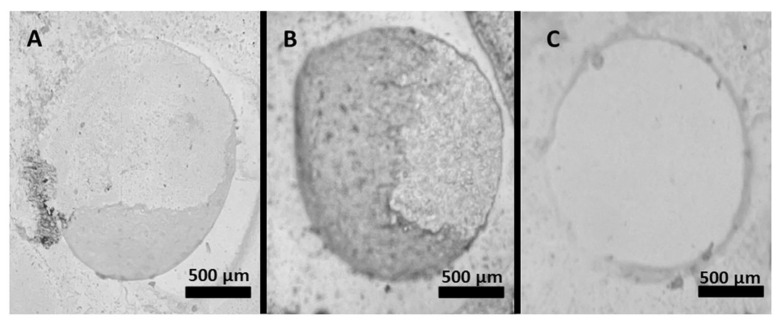
Representative stereomicroscope images (40× magnification) of specimens from the microshear bond strength test, illustrating (**A**) cohesive, (**B**) mixed, and (**C**) adhesive failure modes. Scale bars = 500 µm.

The distribution of failure types in adhesive resin cement samples is presented in Figure 2, and representative stereomicroscope images are shown in Figure 3. Adhesive failure, defined as separation at the resin–ceramic interface, was the predominant failure mode across all groups and was more frequent in specimens that did not undergo thermocycling. The highest incidence of adhesive failure was observed in the IPS e.max CAD material without thermal cycling, while the lowest incidence of adhesive failure was observed in the Amber Mill material with thermal cycling. Cohesive failure, known as fractures within the resin composite itself, was least observed in the IPS emax CAD group that did not undergo thermal cycling, while the highest incidence of cohesive failure was detected in the Amber Mill material that underwent thermal cycling.

## 4. Discussion

This study evaluated the effects of different surface treatments on three CAD-CAM lithium disilicate-based ceramics (AM, VS, IPS) by analyzing surface roughness, water contact angle, and micro-shear bond strength. The findings led to the research hypothesis was not accepted as that different surface treatments and the type of lithium disilicate CAD-CAM ceramic material significantly influenced the surface roughness, wettability, and bond strength values, and that thermal aging altered these effects.

Although direct microstructural characterization (SEM/XRD/XPS) was not included, previous studies have demonstrated that hydrofluoric acid etching selectively dissolves the glassy matrix, revealing elongated lithium disilicate crystals and increasing micromechanical interlocking [20]. In contrast, laser irradiation may induce localized melting and recrystallization, modifying the crystalline phase and surface energy, which could explain the altered wettability and bonding response observed, especially at higher power settings. These findings suggest that the microstructural response to each surface treatment is material-specific, governed by the distribution and composition of crystalline and glassy phases.

In AM ceramics, neither surface treatment nor thermal aging produced significant differences between groups. In vs. ceramics without thermal aging, significant variations were observed compared to the control group: hydrofluoric acid etching reduced surface roughness to the lowest level, whereas sandblasting and both 2 W and 3 W laser applications increased it (*p* < 0.05). These material-specific responses can be explained by differences in microstructure. In vs. (ZLS), acid-resistant zirconia nanoparticles are dispersed within a silica-based glass matrix, limiting hydrofluoric acid penetration and reducing micro-retentive feature formation. Consequently, hydrofluoric acid etching may even decrease surface roughness while increasing wettability, and thermal cycling further equalizes surface differences among vs. groups [16,21]. Although hydrofluoric acid etching is generally expected to enhance surface wettability, in the present study it produced comparable or slightly higher contact angle values after aging particularly in Amber Mill indicating that its effect may vary depending on the ceramic composition and the proportion of the glassy phase. This finding aligns with previous studies reporting that HF etching can alter surface energy and wettability in a composition dependent manner [13,20]. In contrast, IPS (LDS) contains a higher proportion of etchable glassy matrix (approximately 70%), making it more susceptible to hydrofluoric acid-induced selective dissolution, which produces greater surface roughness compared to sandblasting or laser treatment [21,22,23]. AM (NLD) exhibits a fine and uniform crystalline network, particularly after hydrofluoric acid etching, consistent with previous observations of NLD ceramics [5]. Controlled erbium laser irradiation (Er:YAG, Er,Cr:YSGG) can modify ceramic surfaces while minimizing over-ablation, although hydrofluoric acid etching generally produces superior micromechanical retention [17]. Compositional variations, such as zirconia reinforcement in VS, influence microstructure and explain the differing responses of these ceramics to identical surface treatments [24].

Sandblasting and laser irradiation, on the other hand, primarily create micromechanical retention through superficial abrasion or ablation, often resulting in a more uniform and less aggressive surface topography [25]. In the intermaterial comparison, AM generally had the highest roughness values, while IPS had the lowest (*p* < 0.05). This trend is consistent with previous findings indicating that AM, due to its unique microstructure containing quartz and larger lithium disilicate crystals, exhibits higher baseline and post-treatment roughness compared to IPS [26]. The effect of thermal aging on roughness was limited and only significant in certain laser-treated groups, aligning with previous reports that aging causes minor surface relaxation rather than pronounced topographic alterations [22,27].

Water contact angle values were influenced by both ceramic type and surface treatment. Low-power laser irradiation (2 W) improved wettability, while high-power laser (3 W) produced smoother, glazed-like surfaces with increased hydrophobicity [15,22]. Thermal cycling generally increased contact angles, particularly in control and sandblasting groups, which may be due to surface reorganization or contamination reducing surface energy [18]. Between materials, vs. exhibited lower water contact angle values (greater hydrophilicity) than AM and IPS, a finding likely related to the presence of zirconia reinforcement [24,26].

Micro-shear resistance values showed significant differences depending on the surface treatment and material type, which agrees with recent studies showing that both micromechanical retention and chemical surface alterations are key to optimizing resin–ceramic bonding [26,28]. Control groups yielded the lowest microshear bond strength values across all materials; acid etching, sandblasting, and laser applications provided significantly higher resistance compared to the control group, consistent with reports that micro topographical changes enhance surface energy and improve resin infiltration [29]. In AM ceramics, acid etching, laser 2 W, and laser 3 W applications produced higher microshear bond strength values than sandblasting and control groups; sandblasting, however, yielded lower values than laser 2 W, which may be related to differences in particle embedment and subsurface microcracks affecting adhesion. Additionally, the microshear bond strength may be impacted by the sand particle size, pressure, and probe’s distance [30,31]. Sandblasting can cause excessive cavities or material loss on the ceramic surface, which lowers the material’s flexural strength [32,33,34,35]. Similarly, in vs. ceramics, the control group showed lower resistance than all other surface treatments; acid etching yielded the highest microshear bond strength values, while sandblasting and laser applications remained at an intermediate level (*p* < 0.05), aligning with findings that ZLS ceramics still benefit from hydrofluoric acid etching despite zirconia’s relative acid resistance [24,26]. In IPS ceramics, acid etching provided the highest microshear bond strength, while the 3 W laser also produced high values; sandblasting and the 2 W laser showed lower strengths, supporting evidence that hydrofluoric acid selectively dissolves the glassy matrix in lithium disilicate, generating optimal microretentive patterns [20]. Clinically, each surface treatment has its own strengths and limitations. Hydrofluoric acid etching provides the most effective micromechanical retention but poses safety risks and requires careful isolation. Laser treatment offers a safer, more controlled option with good bonding potential, though its high cost and limited availability may limit use. Airborne-particle abrasion is simple and accessible but can cause surface flaws or microcracks if overused. Therefore, the choice of surface treatment should balance effectiveness, safety, and practicality according to the ceramic type and clinical situation. When the materials were compared, IPS’s microshear bond strengths were generally higher than vs. and AM, while AM’s strengths were the lowest. It is observed in recent comparative studies and attributed to differences in crystalline phase content and microstructure [26]. Thermal cycling increased microshear bond strength values, particularly in the control and sandblasting groups; microshear bond strength values of non-thermally treated samples were often lower than those of thermally treated samples (*p* < 0.05), possibly due to microstructural relaxation or improved interfacial adaptation during thermal stress [22]. Failure mode analysis in this study showed that adhesive failure was the predominant type across all groups, particularly in non-thermocycled specimens, indicating that the interface between the resin cement and ceramic surface remains the weakest link under initial conditions. This was most evident in IPS without thermal cycling, which also presented the highest incidence of adhesive failures. However, after thermocycling, the proportion of adhesive failures decreased, and cohesive or mixed failures became more frequent, particularly in AM ceramics. The higher occurrence of cohesive failures in aged AM specimens may be related to its distinct microstructure, which appears to maintain stronger resin–ceramic adhesion under thermal stress. These results align with recent studies: Detogni et al. observed that adhesive and mixed failures were prevalent in disilicate glass-ceramics bonded with universal cements after thermocycling [21]. It should be noted that the failure analysis in this study was limited to stereomicroscopy at 40× magnification. Although this approach allows reliable qualitative classification of adhesive, cohesive, and mixed failures, it does not provide the high-resolution microstructural detail obtainable with scanning electron microscopy. Pilecco et al. reported that loss of adhesion severely compromises fatigue behavior of lithium disilicate restorations, especially when adhesive failures occur in central bonding areas under aging [36]. Also, the work by Unalan Degirmenci et al. on resin nanoceramics showed that aging (thermocycling & UV) increases surface roughness and shifts failures toward adhesive types [23]. Moreover, a comparative tensile bond strength study of different lithium disilicate ceramics after artificial aging found that mixed failures were common in groups that maintained higher bond strength, while adhesive failures dominated in weaker bonded groups [37]. Overall, the distribution of failure types in our work supports the bond strength findings, emphasizing that both ceramic type and aging conditions influence the durability and mechanism of resin–ceramic adhesion.

These findings indicate that the microstructure of each ceramic determines its response to surface treatments. Hydrofluoric acid etching was most effective for IPS and beneficial for VS, whereas laser treatment was advantageous for AM ceramics. Although thermal aging had limited influence, it could enhance bond strength under certain conditions; thus, surface treatment should be selected according to the ceramic type.

This study isolated the effects of ceramic type, surface treatment, and thermal aging under controlled in vitro conditions. A single self-adhesive resin cement and a standardized thermocycling protocol (10,000 cycles between 5 °C and 55 °C, following the methodology proposed by Gale and Darvell (1999)) [18] were used to ensure methodological consistency and comparability with previous studies. However, as an in vitro study, it does not fully reproduce intraoral factors such as pH changes, mechanical stress, and biofilm formation. The absence of microstructural or compositional analyses (e.g., SEM, XRD, XPS) also limits interpretation. Future studies combining such analyses with long-term clinical validation are recommended to better relate microstructure to surface roughness, wettability, and bond strength.

## 5. Conclusions

Within the limitations of the study, the following conclusions were drawn:

(1) The aim of this study to evaluate the effects of different surface treatments and thermocycling on the surface roughness, water contact angle, and microshear bond strength of three CAD/CAM lithium disilicate-based ceramics was successfully achieved.
(2) Hydrofluoric acid etching resulted in the highest surface roughness and microshear bond strength values for all tested ceramics, confirming its effectiveness in promoting micromechanical interlocking.
(3) Laser irradiation at 2 W produced moderate improvements in surface characteristics and bond strength, while higher laser power (3 W) and airborne-particle abrasion were less effective and occasionally caused surface irregularities.
(4) Water contact angle analysis revealed that hydrofluoric-acid-treated specimens exhibited the most hydrophilic surface, whereas laser and sandblasted groups showed higher contact angles, indicating reduced wettability.
(5) Thermocycling induced moderate but statistically detectable changes in surface characteristics, slightly reducing roughness differences in Vita Suprinity and increasing contact angle values, while bond strength remained stable or showed slight increases in some groups. These findings suggest limited hydrothermal effects rather than significant deterioration after aging.
(6) Among the tested ceramics, IPS e.max CAD generally demonstrated higher microshear bond strength compared with Vita Suprinity and Amber Mill, which may be related to its crystalline content and microstructural characteristics.
(7) From a clinical perspective, hydrofluoric acid etching remains the most effective surface treatment method for achieving durable adhesion between resin cements and lithium disilicate-based ceramics. However, laser irradiation at optimized parameters may serve as a viable alternative when hydrofluoric acid application is contraindicated.

## Figures and Tables

**Table 1:** Description and composition of the lithium disilicate-based CAD/CAM glass-ceramic materials used in this study.

Material	Type/Definition	Manufacturer (Country)	Composition (wt%)	Lot No.
**IPS e.max CAD**	Lithium disilicate glass-ceramic	Ivoclar Vivadent, Schaan, Liechtenstein	SiO_2_ 57–80; Li_2_O 11–19; K_2_O 0–13; P_2_O_5_ 0–11; ZrO_2_ 0–8; ZnO 0–8; others + coloring oxides 0–12	YB5od4WZ
**Vita Suprinity PC**	Zirconia-reinforced lithium silicate glass-ceramic	VITA Zahnfabrik, Bad Säckingen, Germany	SiO_2_ 56–64; Li_2_O 15–21; ZrO_2_ 8–12; P_2_O_5_ 3–8; K_2_O 1–4; Al_2_O_3_ 1–4; CeO_2_ 0–4; pigments 0–4	EC4S010109 89100
**Amber Mill**	Nano-lithium disilicate glass-ceramic	HASS Bio, Gangwon-do, Republic of Korea	SiO_2_, Li_2_O, K_2_O, MgO, Al_2_O_3_, P_2_O_5_, and other oxides	EBE05PD1101

**Table 2:** Surface roughness (Ra, µm) values of lithium disilicate-based CAD/CAM ceramics after different surface treatments and thermocycling.

Surface Treatment	Amber Mill—Non-Aged	Amber Mill—Aged	Vita Suprinity—Non-Aged	Vita Suprinity—Aged	IPS e.max CAD—Non-Aged	IPS e.max CAD—Aged
**Control (no treatment)**	1.04 ± 0.17 A a	0.96 ± 0.13 A a	0.90 ± 0.19 A a	0.72 ± 0.27 A a	0.69 ± 0.16 B b	0.68 ± 0.23 B b
**Hydrofluoric acid etching**	0.87 ± 0.14 A a	1.08 ± 0.21 A a	0.47 ± 0.20 B b	0.59 ± 0.23 B a	0.90 ± 0.12 A a	0.88 ± 0.09 A a
**Airborne particle abrasion**	0.88 ± 0.09 A a	0.99 ± 0.07 A a	0.77 ± 0.14 B a	0.72 ± 0.12 B a	0.60 ± 0.08 B b	0.58 ± 0.15 B b
**Er,Cr:YSGG laser 2 W**	0.93 ± 0.12 A a	0.93 ± 0.10 A a	0.91 ± 0.21 A a	0.63 ± 0.27 B a	0.68 ± 0.13 B b	0.70 ± 0.13 B b
**Er,Cr:YSGG laser 3 W**	0.96 ± 0.09 A a	0.78 ± 0.38 AB a	0.84 ± 0.20 AB a	0.87 ± 0.33 AB a	0.61 ± 0.16 B b	0.56 ± 0.14 B b

Different uppercase letters indicate statistically significant differences within the same row (*p* < 0.05). Lowercase letters indicate significant differences within the same column of each LDS ceramic.

**Table 3:** Water contact angle (°) values of lithium disilicate-based CAD/CAM ceramics after different surface treatments and thermocycling.

Surface Treatment	Amber Mill—Non-Aged	Amber Mill—Aged	Vita Suprinity—Non-Aged	Vita Suprinity—Aged	IPS e.max CAD—Non-Aged	IPS e.max CAD—Aged
Control	61.0 ± 4.7 B a	63.4 ± 2.1 B c	47.8 ± 1.8 D d	61.4 ± 4.2 B a	53.4 ± 3.1 C c	70.0 ± 1.4 A a
Hydrofluoric acid etching	58.6 ± 3.4 B b	70.8 ± 1.6 A b	52.0 ± 2.9 C c	52.8 ± 1.9 C c	60.4 ± 2.3 B b	62.6 ± 2.0 B b
Airborne particle abrasion	62.2 ± 2.3 B a	68.4 ± 2.7 A b	54.0 ± 1.9 C c	57.2 ± 3.1 C b	56.8 ± 2.2 C c	65.0 ± 2.4 A a
Er,Cr:YSGG laser 2 W	54.8 ± 2.2 C b	62.8 ± 5.2 A c	58.8 ± 2.6 B b	66.6 ± 2.1 A a	66.2 ± 1.8 A a	69.0 ± 2.6 A a
Er,Cr:YSGG laser 3 W	63.0 ± 3.2 C a	74.4 ± 1.3 A a	65.6 ± 1.5 C a	63.2 ± 2.4 C ab	62.8 ± 2.2 C b	70.8 ± 2.4 B a

Different uppercase letters indicate statistically significant differences within the same row (*p* < 0.05). Lowercase letters indicate significant differences within the same column of each LDS ceramic.

**Table 4:** Microshear bond strength (MPa) of lithium disilicate-based CAD/CAM ceramics after different surface treatments and thermocycling.

Surface Treatment	Amber Mill—Non-Aged	Amber Mill—Aged	Vita Suprinity—Non-Aged	Vita Suprinity—Aged	IPS e.max CAD—Non-Aged	IPS e.max CAD—Aged
Control	1.95 ± 0.66 E c	6.87 ± 2.40 B c	3.05 ± 1.51 D c	7.21 ± 2.67 B a	7.12 ± 1.34 C b	9.50 ± 1.43 A b c
Hydrofluoric acid etching	9.59 ± 1.98 B a	13.58 ± 3.18 A a	10.18 ± 1.80 B a	7.38 ± 1.61 C a	10.86 ± 1.51 AB a	12.05 ± 2.25 A a
Airborne particle abrasion	7.80 ± 2.03 B b	11.11 ± 2.86 A b	7.80 ± 1.79 B b	4.75 ± 1.89 C b	6.70 ± 1.40 B b	10.18 ± 1.67 A b
Er,Cr:YSGG laser 2 W	9.76 ± 2.44 B a	11.96 ± 2.92 A b	7.55 ± 3.25 C b	7.89 ± 3.05 C a	6.62 ± 1.61 D b	8.65 ± 1.46 C c
Er,Cr:YSGG laser 3 W	9.76 ± 2.95 A a	11.45 ± 2.72 A b	8.14 ± 2.03 B b	8.48 ± 1.96 B a	10.26 ± 1.63 C a	12.22 ± 2.14 A a

Different uppercase letters indicate statistically significant differences within the same row (*p* < 0.05). Lowercase letters indicate significant differences within the same column of each LDS ceramic.

## Data Availability

The original contributions presented in this study are included in the article. Further inquiries can be directed to the corresponding author.

## Abbreviations

NLD	Nano lithium disilicate
ZLS	Zirconia-reinforced lithium silicate
LD	Lithium Disilicate
AM	Amber Mill
VS	Vita Suprinity
IPS	IPS e.max CAD
